# Annotated type catalogue of the Amphibulimidae (Mollusca, Gastropoda, Orthalicoidea) in the Natural History Museum, London

**DOI:** 10.3897/zookeys.138.1847

**Published:** 2011-10-19

**Authors:** Abraham S.H. Breure, Jonathan D. Ablett

**Affiliations:** 1 Netherlands Centre for Biodiversity Naturalis, P.O. Box 9517, Leiden, the Netherlands; 2 Natural History Museum, Division of Higher Invertebrates, London, SW7 5BD, UK

**Keywords:** Amphibulimidae, types, biohistory

## Abstract

The type status is described of 39 taxa classified within the family Amphibulimidae (superfamily Orthalicoidea) and kept in the London museum. One taxon, *Bulimus elaeodes* Pfeiffer, 1853, is removed to the Strophocheilidae. Lectotypes are designated for *Bulimus adoptus* Reeve, 1849; *Bulimus (Eurytus) eros* Angas, 1878; *Helix onca* d'Orbigny, 1835; *Amphibulima pardalina* Guppy, 1868. The type status of the following taxon is changed to lectotype in accordance with Art. 74.6 ICZN: *Strophocheilus (Dryptus) jubeus* Fulton, 1908.

As general introduction to this and following papers on Orthalicoid types in the Natural History Museum, a brief history of the London collection is given and several examples of handwriting from different authors are presented.

## Introduction

Annotated catalogues of type specimens are an important source of information on the primary types that constitute the basis of a stable taxonomy for a group. For the superfamily Orthalicoidea, with more than 1750 available taxa names, this has partly been achieved by the papers of Zilch (1971, 1972), Breure (1975, 1976, 2011), Neubert and Janssen (2004) and Köhler (2007). Breure (1979) and Breure and Schouten (1985) listed all the primary type material for this group, known at that time. During the course of an ongoing revision of the Orthalicoidea, which now also includes phylogenetic research (Breure et al. 2010, Breure and Romero in press), it became evident that a re-study of types and a documentation up to present-day standards were needed to ensure a stable taxonomy at the species level. This paper is a first contribution presenting the type material of the Orthalicoidea in the collection of the Natural History Museum (hereafter NHM or the Museum), London, United Kingdom, which now houses more than 600 types of nominal taxa from this group. For practical reasons, this paper is dealing with the Amphibulimidae only. However, in the following introduction to the collection and the handwritings found therein, some examples from other families within the Orthalicoidea will be mentioned; these other families will be treated in subsequent papers.

## The collection

The NHM collection is famous for its large amount of primary types of taxa, many of which were described throughout the 19^th^ Century. For a concise general history of the collections, see Stearn (1981: 194–197); for acquisitions up to 1904, see E.A. Smith (1906). Biographical references have largely been obtained from Coan et al. (2011). In the context of this paper, the following acquisitions are important to mention as they contained type material of several taxa dealt with herein.

In 1837 the Trustees of the Museum purchased the collection of William J. Broderip, which probably also contained type material of species he described together with Sowerby (Broderip and Sowerby 1832a, b). However, some of these types came into the Cuming collection (see below), and were further exchanged (see Köhler 2007, Neubert and Janssen 2004). Around the same time, part of the extensive collection made by Lansdown Guilding in the West Indies was acquired at an auction, containing “the actual types or co-types of the various species described by Mr. Guilding” (E.A. Smith 1906: 704). However, none of the taxa described by Guilding pertaining to the Orthalicoidea could be traced during this research.

The collection of Hugh Cuming has been a source for thousands of species descriptions, giving it a unique position in its time-frame. As far as can be traced from the registration books in NHM, several series were purchased during Cuming's lifetime. In 1842 and 1843 the Museum purchased about 1800 specimens collected by Cuming, who lived for several years in Chile and made collecting trips to, among others, parts of South and Central America (Melvill 1895). He not only collected himself, but also gathered specimens from various sources, through exchange, and with the help of various assistants, e.g., Thomas Bland (see Martin 1886), Bourcier (possibly Jules Bourcier, who was at the time French consul to Ecuador; see Beolens and Watkins 2003), his son-in-law Thomas Bridges (Dall 1866), David Dyson (an assistant to Cuming, who collected in the Neotropics; le Tomlin 1945), Nicolas Funck (who was a draftsman to J. Linden and accompanied him during his collecting travels; his third trip was to Venezuela in 1841–1842 and he returned to that country in 1845; see Urban 1902), Gueinzius (possibly Wilhelm Gueinzius, who never travelled in South America, but who exchanged extensively with Eduard Friedrich Poeppig, a German naturalist who spent several years in Brazil, Chile, and Peru; see Urban 1902), Karl Theodor Hartweg (a German botanist who collected extensively in Central America, Ecuador and Colombia (see Urban 1902, Anonymous 2011a), William Lobb (an English plant collector who travelled in South America in 1840–1848; see Shepard 2003), John Miers (an English botanist who lived for some years in Brazil; see Anonymous 2011b), Auguste Sallé (a French malacologist; see Crosse 1897), Louis Joseph Schlim (travelled with J. Linden to Venezuela, New Granada, Jamaica and Cuba from 1841–1844, and together with N. Funck in 1845 to Guadeloupe and Venezuela; see Urban 1902), and Richard Spruce (an English botanist who spent approximately 15 years exploring the Amazon from the Andes to its mouth; see Seaward and Fitzgerald 1996). As far as we could detect none of the lots inspected were found accompanied with a label bearing Cuming's handwriting (see also below). Cuming, “in the most free and liberal manner, opened the collection to the use of (..) conchologists and iconographers as would fall into his views as to the describing and naming of species” (Gray 1868: 726; cf. Gray 1869 where this account on the Cuming collection was re-published, spreading this tale also to the New World). Lovell Reeve and the Sowerby family made extensive use of this opportunity to describe and publish many species and publish series of books, as documented by Petit (2007, 2009). But Cuming also made contacts with continental malacologists, of which Louis Pfeiffer needs a special mention in the context of this paper. According to Neubert and Janssen (2004: 196) “in 1845, a large suite of terrestrial molluscs were exchanged with H. Cuming containing a considerable number of voucher specimens to the important works of (..) L. Pfeiffer”. Gray (1868: 728) says that “Mr. Cuming was in the habit of sending to Dr. Pfeiffer, Reeve, Sowerby, and other describers and figurers of the species certain specimens from his duplicates marked with the same number as that attached to his own specimens; and the determination of the species depended on the accuracy with which these numbers were reported” (see also below and Fig. 5). Although a few cases have been spotted during our revisionary work where an obvious mistake has been made, it has also been possible to match many specimens to the original dimensions or figures given by Pfeiffer, Reeve, and Sowerby. This implies that specimens on which species descriptions were based were often returned by Pfeiffer to Cuming's collection. After the death of Cuming in 1865, the collection was acquired by the Museum in 1866 (E.A. Smith 1906: 710).

In 1844, the Museum obtained the material collected during the surveying voyage of H.M.S. *Fly* along the coasts of New Guinea and Australia (see Jukes 1847 [2011]). The material, collected by the naturalist John MacGillivray, contained several new species of Placostylidae later described by Pfeiffer. Also the material from the surveying voyages of H.M.S. *Herald* and H.M.S. *Pandora*, commanded by Captain Henry Kellett and Lieut. Wood respectively, along the coast of California and the Pacific coast of Central and South America (see Seemann 1853, Samson 1998, Colledge and Warlow 2010) was presented to the Museum shortly afterwards . Several species of Bulimulidae were described on the basis of this material by Edward Forbes (1850).

The collection of Alcide d'Orbigny came to London in 1854 (E.A. Smith 1906: 707). Part of it is based on the specimens collected during his journeys to South America (Gray 1854) and includes most of the specimens dealt with in his “Voyage...” (d'Orbigny 1834–1847) (dates according to Sherborn and Griffin 1934). Many taxa had been briefly described before in d'Orbigny (1835), but the importance of the “Voyage...” was mainly in the elaboration of the localities (see Breure 1973 for localisation in modern geography), and in accurately figuring most of the taxa. Between 1870 and 1886, the collection of Australian material made by George French Angas, containing many types, was donated by him to the Museum (see also Iredale 1959). In the same period the collection of Robert John Lechmere Guppy, an Englishman who lived for many years in Trinidad (see Newton 1917), came to the Museum. The material comprised the type specimens of taxa described by him from various islands in the West Indies. In 1875, the collection of Thomas Lombe Taylor was presented by his widow. Its importance is mainly marked by the many species described by Lovell Reeve in the “Conchologia Iconica” (see also Dance 1986: 170–171). In 1883 the Museum purchased the collection of Jean Baptiste Gassies (see Crosse and Fischer 1884), containing many types of Placostylidae described from New Caledonia. Ten years later the collection of Arthur Morelet came to London after having been bought at an auction by Fulton, a well-known dealer at that time (see Fulton 1920). It contained all the types described by Morelet, including several Bulimulidae from South America.

In 1901 Frederick DuCane Godman presented to the Museum his extensive collection of biological material from Central America. Jointly with Osbert Salvin he was co-editor of a multi-volume encyclopaedia on the natural history of that area, of which the land and freshwater Mollusca were treated by Eduard von Martens (1890–1901). The types of species described by von Martens can be found in the Godman collection. During the years 1902–1904, several type specimens described by James Cox (Placostylidae) and by James Cosmo Melvill and John H. Ponsonby (*Prestonella*) were either purchased or presented. Also type material described by S.I. da Costa and W.K. Weyrauch was presented by these authors to the Museum. Via dealers like H.B. Preston and Sowerby and Fulton, the Museum acquired material that had been either described by these dealers or originated from continental collections (e.g. Grateloup, Rolle).

For a complete understanding of the collection it is also necessary to know the history of its staff. While John Edward Gray was one of the first Keepers of the Zoology Collection (1840–1875), Edgar Albert Smith was certainly the most prominent staff member during the late 19th century; he joined the Museum in 1867 and retired in 1913. After his retirement, the Mollusca Section was formally set up. Guy Coburn Robson (1888–1945) was the first head of section, and had been working on the collections since 1911, when he entered the Museum after study at Oxford and in Naples. He had a particular interest in cephalopods, and published an important monograph in 1931–1936, but also wrote on broader problems of species and variation. When Robson resigned due to ill health in 1935 he was succeeded by George Ivor Crawford, who had studied at Cambridge and worked at the Marine Biological Laboratory in Plymouth. Crawford was followed in 1946 by William James Rees (1913–1967), who was heavily involved in the post-war reconstruction of the galleries and a reorganisation of the collections. Like Robson, he paid particular attention to the cephalopods until he moved to the Coelenterate Section in 1955. The fourth head of the Section was Ian Courtney Julian Galbraith, who was followed by Norman Tebble in 1959 when he transferred to the Bird Section. The heads of section were assisted by J. C. Vickery, who joined as a Boy Attendant in 1897, and finally retired as a Higher Grade Technical Assistant in 1947 (Hindle 1946, Crawford 1967, Stearn 1981).

## Labels, author's handwriting and matching specimens

**Figure 1.**
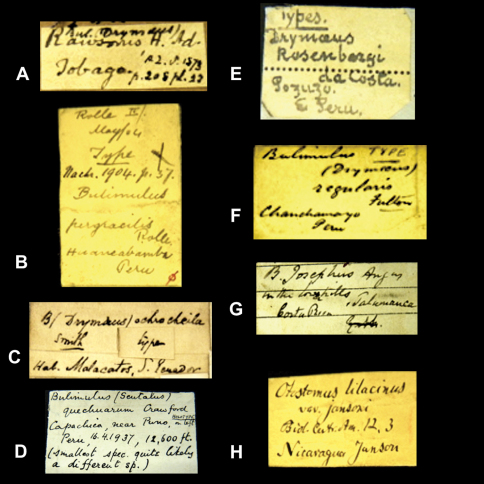
Labels of species bearing the author's handwriting. **A** H. Adams. **B** H. Rolle. **C** E.A. Smith. **D** G.I. Crawford. **E** S.I. da Costa. **F** H.C. Fulton. **G** C.F. Angas. **H** E. von Martens.

**Figure 2.**
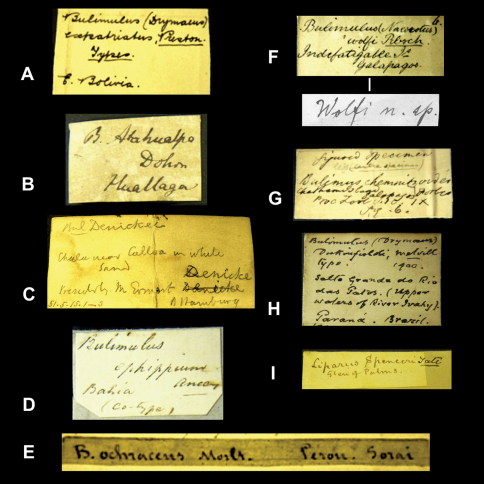
Labels of species bearing the author's handwriting. **A** H.B. Preston. **B** H. Dohrn. **C** J.E. Gray (?). **D** C.M.F. Ancey. **E** A. Morelet. **F** Reibisch. The upper label has possibly been written by P. Reibisch, but it could also have been some else of the Reibisch family (K. Schniebs, pers. comm.). The lower label is in the handwriting of P. Reibisch (courtesy of K. Schniebs). **G** E. Forbes. **H** J.C. Melvill. **I** R. Tate. The author's name has been added in the NHM.

**Figure 3.**
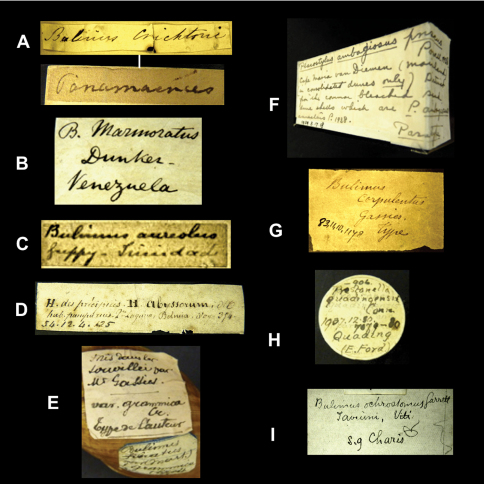
Labels of species bearing the author's handwriting. **A** W.J. Broderip. **B** W. Dunker. **C** R.J.L. Guppy. **D** A. d'Orbigny. **E** H. Crosse. The labels have been glued upon the shell. **F** A.W.B. Powell. The label is glued onto the cardbox. **G** J.B. Gassies. **H** M.W.K. Connolly. **I** A. Garrett.

**Figure 4.**
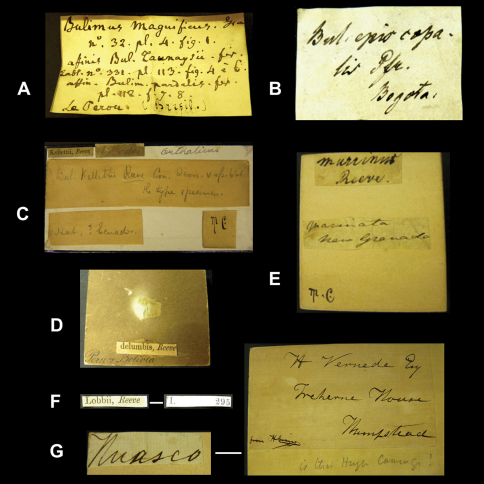
**A–B** Labels of species bearing the author's handwriting. **A** J.P.S. de Grateloup. **B** L. Pfeiffer. **C–F** Labels of species described by L.A. Reeve. **C–D** Taxon name on printed labels. All other information seems to have been added after the arrival of the Cuming collection in NHM (post-1866); note the ambiguous locality information in **D. E** Taxon name in handwriting, probably in Pfeiffer's hand. **F** Two sides of a printed taxon label. *Recto*, the name and author of the species (Index: v, left row, third line from below). *Verso*, part of reference to Table and Species number (Index: vi, right row, third line from below). **G** Locality label probably in handwriting of one of Cuming's assistants. The text on the right-hand side was found in the archive of NHM Mollusca section.

Historical collections are not only a rich source of type material but they also permit us to have a glimpse back in time. Labels and their handwriting are often the sole remnants of work done by malacologists in the past. In the context of this project we came across many labels bearing original handwriting. Although some examples are given elsewhere (e.g. Dance 1966, Zilch 1967, Wood and Gallichan 2008, Breure 2011), it seems useful to present an overview of handwritings we encountered during this research and which we can attribute to authors of taxa (Figs 1–3, 4A–B).

**Figure 5.**
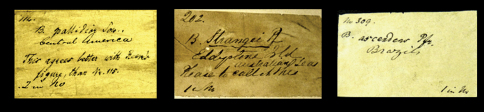
Examples of labels with a reference number in the upper left-hand corner and a text on the lowest line most likely referring to the number of lots under this reference number (e.g., “1 in No.”). Note that the labels all bear the taxon name in Pfeiffers's handwriting (plus additional notes in the left-hand example).

As pointed out above, the Cuming collection is a rich source of material and this also extends to interesting labels. For example there are many examples (Fig. 4B) of labels with Pfeiffer's handwriting, which is quite characteristic and has been published before (Zilch 1967: 36). Although it is difficult to reconstruct the past with an accuracy that rules out any assumptions, the following observations may help to partially explain the way the Cuming collection was dealt with. Cuming himself has rarely left his handwriting on labels (see also Petit 2007: 74). Most of his labels were written by his collectors and his assistants (e.g., Fig. 4G), who wrote an abbreviation for the genus name plus the locality data and a number that apparently was used to check when the determinations came back. On the last line of some labels we have found some unknown reference, e.g. “1 in No.”. Contrary to remarks found on labels added in a 20^th^ Century handwriting, we are not of the opinion that this referred to the number of specimens, but instead to the number of lots that were sent under a given reference number (examples in Fig. 5). The examples also show that Cumingian material was either sent to Pfeiffer for identification and was afterwards returned to London, or Pfeiffer made his identifications during “his frequent trips to London to consult the Cuming collection” (Dance 1986: 122; see also Wheeler 1949: 52).

**Figure 6.**
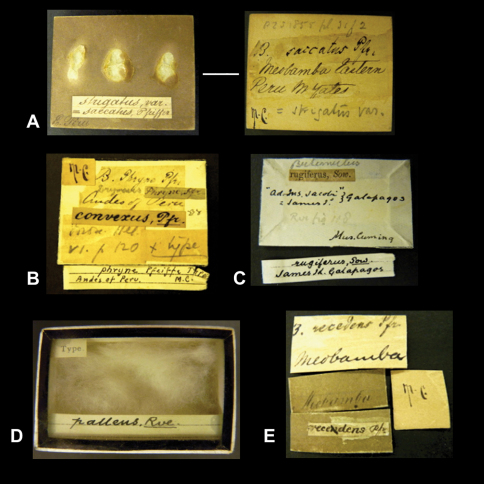
Label types in the Cuming collection. **A** Original wooden tablet. *Recto*, one side showing the places where the shells had been glued, a taxon label written by an assistant, and the locality written in the left-hand corner. *Verso*, Original label glued on the tablet, stating the locality and the taxon name (in this case, in Pfeiffer's handwriting), and notes by subsequent curators. The locality label is probably in the handwriting of Robert Furley Geale, who worked for Cuming as an Assistant for many years (P. Dance, pers. comm.). The characteristic abbreviation “M.C.”, added after the collection had arrived in the NHM in 1866, appears in black ink in the left-hand corner. **B–C** Only the bottom of the cardboard has been preserved to which the labels are still glued. The summarizing label (with text written on lines) is kept as the second label. In **B** the upper taxon label bears Pfeiffer's handwriting; the locality data probably have been written by one of Cuming's assistants. The label “convexus, Pfr.” is possibly in E.A. Smith's handwriting. The label at the bottom in G.I. Crawford's handwriting. In **C** all text in ink is probably by E.A. Smith. **D** The labels are gummed to the bottom of the cardboard box in which the specimens are housed, with a summarizing label on the top side behind the glass lid. **E** Labels which have been soaked off the wooden tablet and which are kept inside an archieval pocket.

There has been some debate in literature about the accuracy of locality labels of Cuming material (Smith 1906: 710–711; Dance 1966: 167–170; Dance 1986: 127–129; Petit 2007: 30). “In many of the specimens, especially those that have not yet been determined or named, the habitat, written on a small paper label, is stuffed into the mouth of the shell” (Gray 1868: 727). Later these labels were gummed to the back of wooden tablets, as Gray (o.c.: 729) writes “I have had the shells of the Cumingian collection placed on [wooden] tablets so that they may be arranged in the same series as the other shells in the British Museum; but each tablet is marked in such a manner that it may be at once distinguished from the rest of the collection, so that there can be no doubt about which are the types or the presumed types of the species described from the collection”. These marks are “M.C.” or “Mus. Cuming” (Figs 6B–C). On the front side, the tablets have been covered with a sheet of gray paper, on which a summary of taxon name and locality data have been added, presumably after the arrival of the collection at the Museum. Around the turn of the century, glass-topped cardboard boxes came into use to house some of the specimens. In the course of the 20^th^ Century, it was decided to start with removing the shells and the labels from these wooden tablets or to cut the bottom of the cardboard boxes, mainly to save space (K.M. Way, pers. commun.). Due to the enormous amount of material, this has only partly been achieved so far. Therefore shells from the Cuming collection can now be found with one of the following ‘label types': (I) the labels are still gummed on one or both sides of wooden tablets (Fig. 6A); (II) the labels are gummed to the bottom of the cardboard box in which the specimens are housed, with a summarizing label on the top side behind the glass lid (Fig. 6D); or (III) only the bottom of the cardboard has been preserved to which the labels are still glued (Figs 6B–C); (IV) the labels have been soaked off and are kept in archival pockets placed together with the specimens in an open box (Fig. 6E).

The specimens figured by Reeve in his ‘Conchologia Iconica' (see also Petit 2009: 46) are never accompanied by written labels from that author (Figs 4C–E); instead, they have small printed labels with the taxon name on one side and the reference to a plate and figure on the other side (‘label type' V: Fig. 4F); the font and position suggests that these labels were cut from a spare index to the ‘Conchologica Iconica'. The shells in these lots can generally be matched to the published figures, as these are very accurate with regard to the shell shape, size and colour (K.M. Way, pers. comm.). However, several instances have been found where lots labelled in the indicated way could not be matched to the original figures; it may have been that also duplicate sets have been labelled with these printed labels. The shells are usually figured in the “Conchologia Iconica” to their actual size, or the figures are accompanied by lines that indicate such size although two additional observations are worthwhile mentioning. While Reeve is known to have generally indicated by a scale bar whenever he figured a shell larger than actual size, some exceptions have been encountered (e.g., Plate XIV). The second observation is related to the way the shells might have been measured. Whenever the shells were elongate in shape with a high height/diameter ratio, the figured specimen always gave a good match. However, when the shell was more globose (viz. a lower height/diameter ratio), the figured specimen only had a good match when it was placed with the aperture downside; thus contrary to more modern practices where shells are always measured perpendicular to the ventral view.

In the collection, labels were found with handwriting that is attributed to the following persons (references to biographical data included): Henry Adams (Crosse and Fischer 1878; Fig. 1A), César-Marie-Félix Ancey (Wood and Gallichan 2008; Fig. 2D), George French Angas (Melvill 1890; Fig. 1G), William John Broderip (Melvill 1890; Fig. 3A), Matthew William Kemble Connolly (le Tomlin 1947; Fig. 3H), George Ivor Crawford (Fig. 1D), Joseph Charles Hippolyte Crosse (Poyard 1898; Fig. 3E), Solomon Israel da Costa (Melvill 1908; Fig. 1E), Wilhelm Dunker (Kobelt 1885; Fig. 3B), Hugh Coomber Fulton (Smith 1906; Fig. 1F), Edward Forbes (Melvill 1890; Fig. 2G), Jean Pierre Sylvestre de Grateloup (Fischer 1862; Fig. 4A), John Edward Gray (Anonymous 1875; Fig. 2C), Robert John Lechmere Guppy (Newton 1917; Fig. 3C), Karl Eduard von Martens (Kobelt 1905; Fig. 1H), James Cosmo Melvill II (Jackson 1930; Fig. 2H), Arthur Morelet (Crosse 1893; Fig 2E), Alcide d'Orbigny (Germain 1933; Fig. 3D), Louis Pfeiffer (Crosse and Fischer 1878; Figs 4B, 5), Rudolp Amandus Philippi (Barros 1904; Fig. 4G), Hugh Berthon Preston (Winckworth 1946; Fig. 2A), Paul Hermann Reibisch (Schniebs 1999; Fig. 2F), Hermann Rolle (Zilch 1967; Fig. 1B), Ralph Tate (Blake 1902; Fig. 2I).

When interpreting possible type material, it is always good practice to check against the original publication (e.g. locality, dimensions, collector). However, when working with historical collections, one cannot always expect the same data that is given in present-day publications, and often one has to investigate with a biohistorical time-frame in mind. In the case of material dating back to the early 19^th^ century, written accounts documenting the history of a collection have vanished in many cases or label handwriting has faded away. And while malacologists like Broderip, Reeve, and Sowerby generally have not left their handwriting in collections (but see Fig. 3A for an exception), it may safely be assumed that they were in contact and may well have swapped material amongst their collections (K.M. Way, pers. comm.). In general, lots originating from older collections, such as the Cuming collection, may not always be accompanied by label data that exactly matches the locality data given in the original publication. Some cases were found where labels have been added during later years, giving a different or broader defined locality than the original label has (Fig. 4D; compare the original published description and the label found in the Cuming collection, with a handwriting that is probably of an assistant during the late 19^th^ century). This may have added in some instances to confusion in subsequent literature about the occurrence and distribution of a taxon.

## Methods

When assessing possible type material, the following criteria have been applied: (a) the authorship and the locality fit with the original description (but see note above on the differences which may occur between published locality data and those on labels); (b) alleged type material is in accordance with the established understanding of the taxon. In order to fulfill the requirements of article 74 of the International Code of Zoological Nomenclature (ICZN), any lectotype designations herein are to be understood as to have the sole purpose to fix the status of these specimens as the sole name-bearing type of that nominal taxon, to ensure the name's proper and consistent application, even when this is not explicitly done in every single case but abbreviated as “lectotype designation”. Lectotypes designated herein are made using the following criteria, in order of preference: (1) the relevant specimen was figured in the original description, or in subsequent revisionary works; (2) if no original figure was published, a specimen was selected that matches as closely as possible the measurements given in the original description. If it is known that the original collection has been destroyed (e.g., Pfeiffer, Strebel; teste Dance 1966), and specimens have been found with labels in the original author's handwriting or originating from the original author, these are herein treated as possible syntypes.

For each taxon the original publication—in which the taxon was proposed—is mentioned, as well as papers in which reference is made to the **Type material.** The type locality is quoted from the original publication in the original wording and language, with clarifying notes between square brackets. The name of the collector, if given in the original paper, is only mentioned (in italics) if it might give a clue about the type status of material present in the collection. The text of the original, or oldest, label is quoted, together with information from subsequent labels if containing information necessary for a correct interpretation. All labels have been photographed and are figured for future historic reference. The original dimensions are quoted, if necessary transferred to mm (see Stöver 1986; see also Rowlett 2004). Dimensions of the type specimens have been taken with a digital caliper, using the methods figured by Breure (1974: fig. 2); measurements up to 10 mm have an accuracy of 0.1 mm, those above 10 mm are accurate to 0.5 mm. Due to improvements in accuracy of calipers, the measurements given herein are in several cases slightly different from those reported by Breure (1978), Breure and Eskens (1981) and Breure and Schouten (1985). Comparing the current measurements to those quoted from the original publication, one should be aware that the diameter especially may have been measured differently. In the case of syntypes, only the largest specimen has been measured. Under type material the NHM-registration numbers are given; if specimens from different localities are present, the order of the lots corresponds with the information of the different labels. The number of specimens originally available, if quoted by the original author, are mentioned under **Remarks.** Remarks are further given to describe any individual characteristics of the type specimens or any other details of the type lot. The current systematic position is given, following the generic scheme of Breure (1979) and the familiar arrangement of Breure et al. (2010) and Breure and Romero (in press).

Abbreviations used for depositories of material are: NHM, Natural History Museum, London, U.K.; RMNH, Netherlands Centre for Biodiversity Naturalis, Leiden, the Netherlands; SMF, Natur-Museum Senckenberg, Frankfurt am Main, Germany; ZMB, Zoologisches Museum, Humboldt Universität, Berlin, Germany. Other abbreviations used are: **/** end of line in cited text; coll., collection; D, diameter; H, shell height; M.C., Cuming collection; leg., *legit*, collected; W, number of whorls.

## Systematics

### Systematic list of taxa arranged in generic order

This systematic list follows the generic classification from Breure (1979), amended as proposed by Breure and Romero (in press), and unpublished data from the senior author; genera are presented in alphabetical order. As for some genera no phylogenetic data have been obtained yet (e.g. *Dryptus*), their familiar relationship remains tentative until a more satisfactory arrangement can be presented.

### Family Amphibulimidae P. Fischer, 1873

***Amphibulima*** Lamarck, 1805

*Amphibulima pardalina* Guppy, 1868.

***Dryptus*** Albers, 1860

*Dryptus adoptus* Reeve, 1849; *guerini* Pfeiffer, 1846; *Dryptus jubeus* Fulton, 1908; *Dryptus marmoratus* Dunker, 1844.

***Pellicula*** P. Fischer, 1856

*Pellicula appendiculata* Pfeiffer, 1847.

***Plekocheilus (Aeropictus)*** Weyrauch, 1967

*Plekocheilus (Aeropictus) cathcartiae* Reeve, 1849; *Plekocheilus (Aeropictus) dissimulans* Preston, 1909; *Plekocheilus (Aeropictus) latilabris* Pfeiffer, 1855; *Plekocheilus (Aeropictus) quadricolor* Pfeiffer, 1848; *Plekocheilus (Aeropictus) scytodes* Pfeiffer, 1853; *veranyi* Pfeiffer, 1848; *Plekocheilus (Aeropictus) zilchi* Breure, 1977.

***Plekocheilus (Eudolichotis)*** Pilsbry, 1896

*Plekocheilus (Eudolichotis) aurissciuri* Guppy, 1866; *Plekocheilus (Eudolichotis) dillwynianus* Pfeiffer, 1853; *Plekocheilus (Eudolichotis) lacerta* Pfeiffer, 1855; *Plekocheilus (Eudolichotis) otostomus* Pfeiffer, 1855; *Plekocheilus (Eudolichotis) perdix* Pfeiffer, 1848.

***Plekocheilus (Eurytus)*** Albers, 1850

*Plekocheilus (Eurytus) auriformis* da Costa, 1904; *Plekocheilus (Eurytus) bruggeni* Breure, 1978; *Plekocheilus (Eurytus) castaneus* Pfeiffer, 1845; *Plekocheilus (Eurytus) corticosus* Sowerby, 1895; *Plekocheilus (Eurytus) doliarius* da Costa, 1898; *Plekocheilus (Eurytus) episcopalis* Pfeiffer, 1855; *Plekocheilus (Eurytus) eros* Angas, 1878; *Plekocheilus (Eurytus) lamarckianus* Pfeiffer, 1848; *Plekocheilus (Eurytus) onca* d'Orbigny, 1835; *Plekocheilus (Eurytus) piperitus* Sowerby, 1833; *Plekocheilus (Eurytus) pulicarius* Reeve, 1848; *Plekocheilus (Eurytus) rhodocheilus* Reeve, 1848; *Plekocheilus (Eurytus) roseolabrum* E.A. Smith, 1877; *Plekocheilus (Eurytus) superstriatus* Sowerby, 1833; *Plekocheilus (Eurytus) taylorianus* Reeve, 1849.

***Plekocheilus (Plekocheilus)*** Guilding, 1828

*Plekocheilus (Plekocheilus) linterae* Sowerby, 1890; *Plekocheilus (Plekocheilus) loveni* Pfeiffer, 1848; *Plekocheilus (Plekocheilus) plectostylus* Pfeiffer, 1848; *Plekocheilus (Plekocheilus) speciosus* Pfeiffer, 1855; *Plekocheilus (Plekocheilus) taquinensis* Pfeiffer 1855.

### Alphabetic list of taxa by species name

#### Bulimus adoptus

Reeve, 1849

http://species-id.net/wiki/Bulimus_adoptus

Figs 7A–B, 7i

Bulimus adoptus Reeve 1849 [1848–1850]: pl. 82 fig. 608.

##### Type locality.

“Banks of the Orinoco”.

##### Label. 

“Venezuela”. M.C. label type V.

##### Dimensions. 

Not given; lectotype H 83.3, D 42.8, W 5.6.

##### Type material.

NHM 20100517.1–3, lectotype and two paralectotypes (Cuming coll.).

##### Remarks. 

The specimen figured by Reeve has been traced in the collection and is here designated lectotype (design. n.). The specimen has been damaged several times during life-time and the shell is slightly deformed. The synonymisation of this taxon with *Dryptus funckii* (Nyst, 1843) by Pilsbry (1895 [1895–1896]) is here tentatively retained.

##### Current systematic position.

Amphibulimidae, *Dryptus funckii* (Nyst, 1843).

#### Succinea appendiculata

Pfeiffer, 1847

http://species-id.net/wiki/Succinea_appendiculata

Figs 10A–B, 10i

Succinea appendiculata  Pfeiffer 1847:146.

##### Type locality. 

“insula Guadeloupe”.

##### Label. 

“Guadeloupe”; taxon label in Pfeiffer's handwriting. M.C. label type III.

##### Dimensions. 

“Long. 14, diam. 9 mill.”; figured specimen H 14.2, D 10.0, W 1.9.

##### Type material.

NHM 20110303, three syntypes (Cuming coll.).

##### Remarks. 

The specimens are slightly damaged due to the thinness of the shells.

##### Current systematic position.

Amphibulimidae, *Pellicula appendiculata* (Pfeiffer, 1847).

#### Strophocheilus (Eurytus) auriformis

da Costa, 1904

http://species-id.net/wiki/Strophocheilus_auriformis

Figs 14A–C, 14i

Strophocheilus (Eurytus) auriformis da Costa 1904: 5, pl. 1 fig. 1.
Plekocheilus (Eurytus) auriformis  (da Costa); Breure 1979: 29.

##### Type locality. 

“Bogotá, Colombia”.

##### Label. 

“Bogata, Colombia”; in da Costa's handwriting.

##### Dimensions. 

“Long. 74, diam. 38 mm”; holotype H 74.0, D 38.6, W 5.1.

##### Type material. 

NHM 1907.11.21.112, holotype (coll. da Costa).

##### Remarks. 

Da Costa (1904) mentions that “only one specimen has been obtained”.

##### Current systematic position.

Amphibulimidae, *Plekocheilus (Eurytus) auriformis* (da Costa, 1904).

#### Plekocheilus aurissciuri

Guppy, 1866

http://species-id.net/wiki/Plekocheilus_aurissciuri

Figs 27A–C, 27ii

Plekocheilus aurissciuri Guppy 1866: 51.
Plekocheilus (Eudolichotis) aurissciuri  Guppy; Breure 1979: 33.

##### Type locality.

[Trinidad].

##### Label.

“Trinidad”; in Guppy's handwriting.

##### Dimensions.

“Height 1.65 inch, greatest breadth 0.7 inch”; figured specimen H 36.7, D 15.6, W 5.3.

##### Type material. 

NHM 1866.1.3.6, nine syntypes (ex Guppy).

##### Remarks.

This material was mentioned as NHM 1975309 in error by Breure (1979).

##### Current systematic position.

Amphibulimidae, *Plekocheilus (Eudolichotis) aurissciuri* Guppy, 1866.

#### Plekocheilus (Eurytus) bruggeni

Breure, 1978

http://species-id.net/wiki/Plekocheilus_(Eurytus)_bruggeni

Figs 18D–F, 18ii

Plekocheilus (Eurytus) bruggeni Breure 1978: 9, pl. 6 figs 5–7; Breure 1979: 29.

##### Type locality.

“Peru, Dept. Pasco, Huancabamba”.

##### Label. 

“Huancabamba, Peru”.

##### Dimensions.

“H 39.0, D 19.5 [mm]”; holotype H 39.0, D 19.5, W 4.5.

##### Type material. 

NHM 1911.11.2.88, holotype; 1911.11.2.89–90, two paratypes (ex Preston).

##### Remarks. 

There is one paratype RMNH 55122.

##### Current systematic position. 

Amphibulimidae, *Plekocheilus (Eurytus) bruggeni* Breure, 1978.

#### Bulimus castaneus

Pfeiffer, 1845

http://species-id.net/wiki/Bulimus_castaneus

Figs 17A–C, 17i

Bulimus castaneus Pfeiffer 1845: 68; Reeve 1848 [1848–1850]: pl. 32 fig. 197.
Plekocheilus (Eurytus) castaneus  (Pfeiffer); Breure 1978: 10 (lectotype designation); Breure 1979: 29.

##### Type locality. 

“Nova Granada; Vegas on the river Quenden”.

##### Label.

“Vegas of the River Quenden”; taxon label in Pfeiffer's handwriting. M.C. label type I.

##### Dimensions. 

“Long. 70, diam. 39 mill.”; lectotype H 69.5, D 47, W 4.6.

##### Type material.

NHM 1975279, lectotype; 1975280, one paralectotype (Cuming coll.).

##### Remarks. 

The lectotype corresponds to the figure given by Reeve (1848).

##### Current systematic position.

Amphibulimidae, *Plekocheilus (Eurytus) castaneus* (Pfeiffer, 1845).

#### Bulimus cathcartiae

Reeve, 1848

http://species-id.net/wiki/Bulimus_cathcartiae

Figs 11A–C, 11i

Bulimus cathcartiae Reeve 1848 [1848–1850]: pl. 42 fig. 265.
Plekocheilus (Aeropictus) cathcartiae Breure 1978: 18, pl. 11 fig. 7 (lectotype designation); Breure 1979: 32; Borrero and Breure 2011: 13, fig. 5S–U.

##### Type locality. 

“New Granada, Prov. Merida”.

##### Label. 

“New Granada”. M.C. label type IV.

##### Dimensions. 

Not given. Lectotype H 45.4, D 26.5, W 4.5.

##### Type material.

NHM 1975288, lectotype; 1975289, four paralectotypes (Cuming coll.).

##### Remarks. 

The specimen figured by Reeve (fig. 265a–b) was designated lectotype by Breure (1978); the top of this shell is slightly damaged. One paralectotype corresponds to fig. 265c. The specimens are accompanied by a label in Pfeiffer's handwriting “Bul. pintadinus Orb.”.

##### Current systematic position.

Amphibulimidae, *Plekocheilus (Aeropictus) cathcartiae* (Reeve, 1848).

#### Bulimus (Eurytus) corticosus

Sowerby III, 1895

http://species-id.net/wiki/Bulimus_(Eurytus)_corticosus

Figs 15A–C, 15i

Bulimus (Eurytus) corticosus Sowerby III 1895: 214, pl. 13 fig. 2.
Plekocheilus (Eurytus) corticosus  (Sowerby); Breure 1978: 11 (lectotype designation); Breure 1979: 30.
Plekocheilus (Eurytus) episcopalis corticosus  (Sowerby); Borrero and Breure 2011: 26, figs 9C, 10D–G.

##### Type locality. 

[Colombia] “Bogota”.

##### Label.

“Bogota”, in da Costa's handwriting.

##### Dimensions. 

“Long. 58, diam. 30 mm.”; lectotype H 58.7, D 30.0, W 4.3.

##### Type material.

NHM 1907.11.21.110, lectotype; 1907.11.21.11, one paralectotype (da Costa coll.).

##### Remarks.

Sowerby (1895) writes “Type in the collection of Mr. S.I. Da Costa”. The shell corresponds to Sowerby's figure.

##### Current systematic position.

Amphibulimidae, *Plekocheilus (Eurytus) episcopalis corticosus* (Sowerby III, 1895).

#### Bulimus dillwynianus

Pfeiffer, 1853

http://species-id.net/wiki/Bulimus_dillwynianus

Figs 27G–I, 27iii

Bulimus dillwynianus Pfeiffer 1853: 258.
Plekocheilus (Eudolichotis) dillwynianus  (Pfeiffer); Breure 1978: 24 (lectotype designation); Breure 1979: 33.

##### Type locality. 

“Andibus Novae Granadae”.

##### Label.

“Andes N. Granada”, taxon label in Pfeiffer's handwriting. M.C. label type IV.

##### Dimensions. 

“Long. 39, diam. 16 1/2 mill.”; lectotype H 39.5, D 18.5, W 5.1.

##### Type material.

NHM 1975144, lectotype; 1975145, two paralectotypes (Cuming coll.).

##### Remarks. 

The type series proves to be somewhat variable in colour pattern. Only the lectotype has a white line as a bordering ‘shadow' to the brown ones.

##### Current systematic position.

Amphibulimidae, *Plekocheilus (Eudolichotis) dillwynianus* (Pfeiffer, 1853).

#### Bulimus (Eurytus) dissimulans

Preston, 1909

http://species-id.net/wiki/Bulimus_(Eurytus)_dissimulans

Figs 13A–D, 13i

Bulimus (Eurytus) dissimulans Preston 1909: 509, pl. 10 fig. 5.
Plekocheilus (Aeropictus) dissimulans  (Preston); Breure 1978: 19, fig. 17 (lectotype designation); Breure 1979: 32.

##### Type locality.

“Merida, Venezuela”.

##### Label. 

“Merida, Venezuela”.

##### Dimensions.

“Alt. 30, diam. maj. 15 mm”; lectotype H 30.0, D 17.0, W 4.2.

##### Type material.

NHM 1914.4.3.37, lectotype; 1912.5.4.20, paralectotype (in alcohol) (ex Preston).

##### Remarks.

The surface of this species is smooth (Fig. 13D), but the axial pattern is unlike other *Plekocheilus* species. It is here tentatively retained under *Plekocheilus (Aeropictus)*.

##### Current systematic position.

Amphibulimidae, *Plekocheilus (Aeropictus) dissimulans* (Preston, 1909).

#### Strophocheilus (Eurytus) doliarius

da Costa, 1898

http://species-id.net/wiki/Strophocheilus_(Eurytus)_doliarius

Figs 16D–E, 16ii

Strophocheilus (Eurytus) doliarius da Costa 1898: 84, fig. 1; Neubert and Janssen 2004: 208, pl. 1 fig. 1.
Plekocheilus (Eurytus) doliarius  (da Costa); Breure 1979: 30.

##### Type locality. 

“Paramba, Ecuador”.

##### Label. 

“Paramba, Ecuador”, in da Costa's handwriting.

##### Dimensions. 

“Long. 58, diam. 41 mm”; lectotype H 58.0, D 41.5, W 4.6.

##### Type material.

NHM 1907.11.21.117, lectotype (da Costa coll.).

##### Remarks. 

Breure (1979) considered this specimen a holotype. Neubert and Janssen (2004) have pointed out that this specimen should be considered a lectotype [Art. 74.6 ICZN], as da Costa did not state on how many specimens his descripotion was based, and addional material has been found in the SMF collection.

##### Current systematic position.

Amphibulimidae, *Plekocheilus (Eurytus) doliarius* (da Costa, 1898).

#### Bulimus episcopalis

Pfeiffer, 1855

http://species-id.net/wiki/Bulimus_episcopalis

Figs 16A–C, 16i

Bulimus epicopalis Pfeiffer 1855d: 115.
Plekocheilus (Eurytus) epicopalis  (Pfeiffer); Breure 1978: 11 (lectotype designation); Breure 1979: 30; Borrero and Breure 2011: 26, figs 10A–C.

##### Type locality.

[Colombia] “Bogota”.

##### Label. 

“New Granada”, taxon label in Pfeiffer's handwriting. M.C. label type IV.

##### Dimensions. 

“Long. 47–58, diam. 22–27 mill.”; lectotype H 58.0, D 33.5, W 4.5.

##### Type material. 

NHM 1953.11.30.1, lectotype; 1953.11.30.2–3, two paralectotypes (Cuming coll.).

##### Current systematic position.

Amphibulimidae, *Plekocheilus (Eurytus) episcopalis epicopalis* (Pfeiffer, 1855).

#### Bulimus (Eurytus) eros

Angas, 1878

http://species-id.net/wiki/Bulimus_(Eurytus)_eros

Figs 20D–F, 20ii

Bulimus (Eurytus) eros  Angas, 1878: 312, pl. 18 figs 6–7.
Plekocheilus (Eurytus) eros  (Angas); Breure 1979: 30.

##### Type locality. 

“Ecuador”.

##### Label. 

“Ecuador”.

##### Dimensions. 

“Alt. 1 inch 5 1/2 lines, diam. 8 lines [H 36.9 D 16.9 mm]”; lectotype H 35.5, D 18.5, W 3.8.

##### Type material. 

NHM 1879.1.21.2, lectotype (ex Angas).

##### Remarks.

Angas did not state on how many specimens his description was based. The label accompanying the specimen reads “the type”; there is, however, no evidence that this was the sole specimen originating from Angas. Therefore the specimen is now designated lectotype (design. n.).

##### Current systematic position. 

Amphibulimidae, *Plekocheilus (Eurytus) eros* (Angas, 1878).

#### Bulimus guerini

Pfeiffer, 1846

http://species-id.net/wiki/Bulimus_guerini

Figs 7C–D, 7ii

Bulimus guerini Pfeiffer 1846: 40.
Dryptus guerini  (Pfeiffer); Breure 1978: 26 (lectotype designation); Breure 1979: 34; Borrero and Breure 2011.

##### Type locality. 

“Neu Granada”.

##### Label. 

“Nova Granada”, taxon label in Pfeiffer's handwriting. See 

##### Remarks.

M.C. label type IV.

##### Dimensions.

“Long. 41, diam. 18 1/2 mill.”; lectotype H 41.0, D 21.7, W 5.2.

##### Type material. 

NHM 1975272, lectotype; 1975273, two paralectotypes, Funck leg. (Cuming coll.).

##### Remarks.

A second label is present, indicating that the specimens have been found at “Caverns of Chachopo / Prov. of Merida N Gr”. Thus the type locality may now be restricted to Venezuela, Edo. Mérida, Chachopo.

##### Current systematic position.

Amphibulimidae, *Dryptus guerini* (Pfeiffer, 1846).

#### Strophocheilus (Dryptus) jubeus

Fulton, 1908

http://species-id.net/wiki/Strophocheilus_(Dryptus)_jubeus

Figs 8A–B, 8i

Strophocheilus (Dryptus) jubeus Fulton 1908: 86, text fig.
Dryptus jubeus  (Fulton); Breure 1979: 34; Borrero and Breure 2011: 8, figs 3A–B.

##### Type locality.

“Capas, Venezuela”.

##### Label. 

“Capas, Venezuela, 2,000 m”.

##### Dimensions. 

“alt. 111, maj. diam. 57 mm”; lectotype H 117.5, D 58.7, W 5+.

##### Type material.

NHM 1905.5.3.1, lectotype, ex Fulton.

##### Remarks. 

Fulton (1908) remarked that he had seen four specimens. This specimen is the only one which is marked “type”; the top is damaged and thus the original shell height must have been larger than quoted above. The holotype designation by Breure (1979) has to be interpreted as lectotype designation (Art. 74.6 ICZN). During their recent revision, Borrero and Breure (2011) compared the type material to that of *Dryptus guerini* (Pfeiffer, 1846), but tentatively retained Fulton's taxon as a separate species.

##### Current systematic position.

Amphibulimidae, *Dryptus jubeus* (Fulton, 1908).

#### Bulimus lacerta

Pfeiffer, 1855

http://species-id.net/wiki/Bulimus_lacerta

Figs 28A–C, 28i

Bulimus lacerta  Pfeiffer, 1855c: 94, pl. 31 fig. 15.
Plekocheilus (Eudolichotis) lacertus  (Pfeiffer); Breure 1978: 26 (lectotype designation).
Plekocheilus (Eudolichotis) lacerta  (Pfeiffer); Breure 1979: 33.

##### Type locality. 

[Brazil] “Para (*Mr. Yates*)”.

##### Label. 

“Para M^r^ Yates”, taxon label in Pfeiffer's handwriting. M.C. label type IV.

##### Dimensions. 

“Long. 33, diam. 14 mill.”; lectotype H 33.5, D 17.0, W 5.2.

##### Type material. 

NHM 1975303, lectotype; 1975304, two paralectotypes, Yates leg. (Cuming coll.).

##### Current systematic position. 

Amphibulimidae, *Plekocheilus (Eudolichotis) lacerta* (Pfeiffer, 1855).

#### Bulimus lamarckianus

Pfeiffer, 1848

http://species-id.net/wiki/Bulimus_lamarckianus

Figs 18A–C, 18i

Bulimus lamarckianus Pfeiffer 1848: 229; Reeve 1848 [1848–1850]: pl. 24 fig. 156.
Plekocheilus (Eurytus) lamarckianus  (Pfeiffer); Breure 1979: 30.
Plekocheilus (Eurytus) coloratus  (Nyst); Breure 1978: 10 (lectotype designation); Borrero and Breure 2011: 32.

##### Type locality. 

“Andes of New Granada, 8000 feet high (*Funck*)”.

##### Label.

“From the Andes of New Granda / 8000 feet high Mr Funck”, taxon label in Pfeiffer's handwriting. M.C. label type IV.

##### Dimensions.

“Long. 62, diam. 32 mill.”; lectotype H 62.4, D 38.8, W [4.9].

##### Type material.

NHM 1975295, lectotype; 1975296, two paralectotypes, Funck leg. (Cuming coll.).

##### Remarks.

The top of the lectotype is damaged.

##### Current systematic position.

Amphibulimidae, *Plekocheilus (Eurytus) coloratus* (Nyst, 1845).

#### Bulimus latilabris

Pfeiffer, 1855

http://species-id.net/wiki/Bulimus_latilabris

Figs 11D–F, 11ii

Bulimus latilabris Pfeiffer 1855b: 7; Pfeiffer 1855 [1854–1860]: 36, pl. 10 figs 1–2.
Plekocheilus (Aeropictus) latilabris  (Pfeiffer); Breure 1978: 20 (lectotype designation); Breure 1979: 32.
Plekocheilus (Aeropictus) succineoides succineoides  (Petit de la Saussaye); Borrero and Breure 2011: 16, fig. 5J–L.

##### Type locality. 

[Colombia] “Santa Fé de Bogota”.

##### Label. 

“New Granada”, added in a later handwriting. See 

##### Remarks.

M.C. label type IV.

##### Dimensions. 

“Long. 49, diam. 26 mill.”; lectotype H 49.0, D 28.5, W 4.0.

##### Type material. 

NHM 1975127, lectotype; 1975141, one paralectotype (Cuming coll.).

##### Remarks.

The material is accompanied by a label signed by E.A. Smith, indicating that the specimen was figured in Pfeiffer (1854–1860) and were considered “types” by him. Since Pfeiffer based himself on Cuming's material for this taxon, the type status is here not questioned despite the fact that a label in Pfeiffer's handwriting is missing.

##### Current systematic position.

Amphibulimidae, *Plekocheilus (Aeropictus) succineoides succineoides* (Petit de la Saussaye, 1840).

#### Bulimus fulminans linterae

Sowerby III, 1890

http://species-id.net/wiki/Bulimus_fulminans_linterae

Figs 25D–F, 25i

Bulimus fulminans linterae Sowerby III 1890: 582, pl. 56 fig. 12.
Plekocheilus (Plekocheilus) blainvilleanus linterae  (Sowerby); Breure 1978: 6 [not fig. 2] (lectotype designation).
Plekocheilus (Plekocheilus) linterae  (Sowerby); Breure 1979: 29; Neubert and Janssen 2004: 214, pl. 1 fig. 4.
Plekocheilus (Plekocheilus) fulminans linterae  (Sowerby); Breure 2009: 27, figs 4A–D, 9A

##### Type locality. 

[Guyana] “Mount Roraima, British Guiana”.

##### Label. 

“Mount Roraima, British Guiana”.

##### Dimensions.

Not given. Lectotype H 43.8, D 23.8, W 4.6.

##### Type material.

NHM 1889.4.25.1, lectotype; 1889.4.25.2, one paralectotype, ex Miss J.E. Linter.

##### Remarks.

Further paralectotype material is in SMF (Neubert and Janssen 2004).

##### Current systematic position.

Amphibulimidae, *Plekocheilus (Plekocheilus) linterae* (Sowerby III, 1890).

#### Bulimus loveni

Pfeiffer, 1848

http://species-id.net/wiki/Bulimus_loveni

Figs 25A–C, 25ii

Bulimus loveni Pfeiffer 1848: 229.
Plekocheilus (Plekocheilus) blainvilleanus loveni  (Pfeiffer); Breure 1978: 6 (lectotype designation).
Plekocheilus (Plekocheilus) loveni  (Pfeiffer); Breure 1979: 29.

##### Type locality. 

“Colonia of Tovar, Venezuela (*Mr. D. Dyson*)”.

##### Label.

“From the Colonia of Tovar Venezuela / Mr D. Dyson”, taxon label in Pfeiffer's handwriting. M.C. label type IV.

##### Dimensions. 

“Long. 42, diam. 20 mill.”; lectotype H 43.5, D 24.0, W 4.3.

##### Type material.

NHM 1975285, lectotype; 1975286, two paralectotype (Cuming coll.).

##### Current systematic position.

Amphibulimidae, *Plekocheilus (Plekocheilus) loveni* (Pfeiffer, 1848).

#### Bulimus marmoratus

Dunker in Philippi, 1844

http://species-id.net/wiki/Bulimus_marmoratus

Figs 9A–B, 9i

Bulimus marmoratus  Dunker in Philippi 1844 [1842–1844]: 157, pl. 2 figs 1–2.
Dryptus marmoratus  (Dunker); Breure 1978: 26 (lectotype designation); Breure 1979: 34; Borrero and Breure 2011: 9.

##### Type locality.

“Brasilia” [sic, Venezuela].

##### Label.

“Venezuela”, label in Dunker's handwriting. M.C. label type IV.

##### Dimensions.

“Alt. 46''', diam. 26''' [H 100.3, D 56.7 mm]”; lectotype H 86.1, D 46.2, W 5.4.

##### Type material.

NHM 1975474, lectotype, ex Dunker (Cuming coll.).

##### Remarks.

The type locality as given in Philippi (1842–1844) is in error, as this species is only known from Venezuela. Apparently Dunker had seen three specimens, as he writes “I owe the figured specimen to the kindness of Consul Mr. Gruner from Bremen, in whose collection there are two additional, identical specimens”. According to Dance (1966) the Dunker collection is in Berlin, with many types in the Cuming collection. The whereabouts of the Gruner collection are unknown to us. Köhler (2007) does not list any type material of this taxon, hence the Cuming collection seem to be the only extant source of material originating from Dunker. The type status of the London specimen is not questioned as it is accompanied by a label in Dunker's handwriting. The specimen, which was chosen lectotype by Breure (1978), is considerably smaller than the original dimensions and does not fit the figure in Philippi (1842–1844).

##### Current systematic position.

Amphibulimidae, *Dryptus marmoratus* (Dunker in Philippi, 1844).

#### Helix onca

d'Orbigny, 1835

http://species-id.net/wiki/Helix_onca

Figs 19A–C, 19i

Helix onca  d'Orbigny 1835: 8.
Bulimus onca  d'Orbigny 1837 [1834–1847]: 295, pl. 30 figs 1–2; Gray 1854: 19.

##### Type locality.

Not given. [Bolivia] “...non loin du dernier point habité de Tutulima” in d'Orbigny 1837 [1834–1847]; see Breure (1973) for precise data.

##### Label.

“Yuracares (Bolivia)”, label in d'Orbigny's handwriting.

##### Dimensions.

“Longit. 62 millim.; latit. 25 millim.”; lectotype H 66.5, D 25.9, W 5.4.

##### Type material.

NHM 1854.12.4.120, lectotype and three paralectotypes (d'Orbigny coll.).

##### Remarks.

The locality on the label corresponds to the type locality of *Helix pentadina* d'Orbigny, 1835, which has been synonymized with *Helix onca* by subsequent authors; the former name has page precedence. The specimen corresponding to d'Orbigny 1837 [1834–1847]: pl. 30 fig. 1 is now selected lectotype (design. n.). According to Gray (1854) the type specimen of *Bulimus pentadinus* d'Obigny is missing.

##### Current systematic position.

Amphibulimidae, *Plekocheilus (Eurytus) pentadinus* (d'Orbigny, 1835).

#### Bulimus otostomus

Pfeiffer, 1855

http://species-id.net/wiki/Helix_onca

Figs 27D–F, 27i

Bulimus otostomus Pfeiffer 1855a: 291; Pfeiffer 1855 [1854–1860]: 31, pl. 8 figs 12–13.
Plekocheilus (Eudolichotis) euryomphalus  (Jonas); Breure 1978: 24 (lectotype designation).
Plekocheilus (Eudolichotis) otostomus  (Pfeiffer); Breure 1979: 33.

##### Type locality.

“Venezuela”.

##### Label.

“Venezuela”, taxon label in Pfeiffer's handwriting. M.C. label type IV.

##### Dimensions.

“Long. 31, diam. 13 mill.”; lectotype H 31.5, D 13.8.

##### Type material.

NHM 1975307, lectotype; 1975308, two paralectotypes (Cuming coll.).

##### Remarks.

The lectotype, corresponding to Pfeiffer's figure, is misshapen and missing the top whorl.

##### Current systematic position.

Amphibulimidae, *Plekocheilus (Eudolichotis) euryomphalus* (Jonas, 1844).

#### Amphibulima pardalina

Guppy, 1868

http://species-id.net/wiki/Amphibulima_pardalina

Figs 10C–D, 10ii

Amphibulima pardalina Guppy 1868: 432.

##### Type locality.

“Dominica”.

##### Label.

No locality on label.

##### Dimensions.

“Long. 20 millim., lat. 11 millim.”; lectotype H 18.9, D 10.6, W 2.6.

##### Type material.

NHM 1874.10.30.7, lectotype, ex Guppy.

##### Remarks.

The lectotype (design. n.) is damaged at the last whorl.

##### Current systematic position.

Amphibulimidae, *Amphibulima pardalina* Guppy, 1868.

#### Bulimus perdix

Pfeiffer, 1848

http://species-id.net/wiki/Bulimus_perdix

Figs 28D–F, 28ii

Bulimus perdix Pfeiffer 1848: 230.
Plekocheilus (Eudolichotis) perdix  (Pfeiffer); Breure 1978: 26, pl. 9 fig. 7 (lectotype designation); Breure 1979: 34; Neubert and Janssen 2004: 222, pl. 1 fig. 7; Köhler 2007: 128, fig. 8.

##### Type locality.

“Agua de Obispo, New Granada (*Funck*)”.

##### Label.

“From Agua de Obispo / New Granada M^r^ Funck”, taxon label in Pfeiffer's handwriting. M.C. label type IV.

##### Dimensions.

“Long. 36, diam. 15 mill.”; lectotype H 33.5, D 17.0, W 5.2.

##### Type material.

NHM 1975305, lectotype; 1975306, two paralectotypes, Funck leg. (Cuming coll.).

##### Remarks.

Further paralectotype material is in SMF (Neubert and Janssen 2004) and ZMB (Köhler 2007).

##### Current systematic position.

Amphibulimidae, *Plekocheilus (Eudolichotis) perdix* (Pfeiffer, 1848).

#### Bulinus piperitus

Sowerby I, 1837

http://species-id.net/wiki/Bulinus_piperitus

Figs 20A–C, 20i

Bulinus piperitus Sowerby I 1837 [1833–1838]: 8, fig. 93; Reeve 1848 [1848–1850]: pl. 16 fig. 96.
Plekocheilus (Eurytus) piperitus  (Sowerby); Borrero and Breure 2011: 48, figs 17G–J.

##### Type locality.

[Peru] “Huallaga”.

##### Label.

“Hualuago [sic] / Peru”. M.C. label type IV.

##### Dimensions.

Not given. Figured specimen H 55.8, D 31.3, W 5.3.

##### Type material.

NHM 1975329, two syntypes (Cuming coll.).

##### Remarks.

The material is accompanied by a taxon label in Pfeiffer's handwriting. A second label indicates that this specimen has probably been figured by Reeve 1848 [1848–1850].

##### Current systematic position.

Amphibulimidae, *Plekocheilus (Eurytus) piperitus* (Sowerby I, 1837).

#### Bulimus plectostylus

Pfeiffer, 1848

http://species-id.net/wiki/Bulimus_plectostylus

Figs 21A–D, 21i

Bulimus plectostylus Pfeiffer 1848: 230.
Plekocheilus (Plekocheilus) plectostylus  (Pfeiffer); Breure 1978: 8 (lectotype designation); Breure 1979: 29.
Plekocheilus (Eurytus) plectostylus  (Pfeiffer); Borrero and Breure 2011: 28, figs 9C, 10O–Q.

##### Type locality.

[Venezuela] “Chachopo, Province of Merida, New Granada (*Funck*)”.

##### Label.

“From Chachopo province of Merida / New Granada M^r^ Funck”, taxon label in Pfeiffer's handwriting. M.C. label type IV.

##### Dimensions.

“Long. 35, diam. 17 mill.”; lectotype H 36.0, D 22.0, W 4.8.

##### Type material.

NHM 1975287, lectotype, Funck leg. (Cuming coll.).

##### Current systematic position.

Amphibulimidae, *Plekocheilus (Eurytus) plectostylus* (Pfeiffer, 1848).

#### Bulimus pulicarius

Reeve, 1848

http://species-id.net/wiki/Bulimus_pulicarius

Figs 22A–C, 22i

Bulimus pulicarius Reeve 1848 [1848–1850]: pl. 42 fig. 267.
Plekocheilus (Eurytus) pulicarius  (Reeve); Breure 1978: 16 (lectotype designation); Breure 1979: 31; Borrero and Breure 2011: 46, figs 14B, 16G–M.

##### Type locality.

“New Granada”.

##### Label.

“New Granada”. M.C. label type V.

##### Dimensions.

Not given. Lectotype H 31.5, D 19.5, W 4.3.

##### Type material.

NHM 1975281, lectotype; 1975282, two paralectotypes (Cuming coll.).

##### Current systematic position.

Amphibulimidae, *Plekocheilus (Eurytus) pulicarius* (Reeve, 1848).

#### Bulimus quadricolor

Pfeiffer, 1848

http://species-id.net/wiki/Bulimus_quadricolor

Figs 11G–I, 11iii

Bulimus quadricolor Pfeiffer 1848: 229; Philippi 1849 [1847–1851]: 37, pl. 8 fig. 4.
Plekocheilus (Aeropictus) quadricolor  (Pfeiffer); Breure 1978: 21 (lectotype designation); Breure 1979: 32; Borrero and Breure 2011: 13, figs 5X–AA.

##### Type locality.

[Venezuela] “Chachopo, Province of Merida, New Granada (*Funck*)”.

##### Label.

“New Granada”, added in a later handwriting. M.C. label type V.

##### Dimensions.

“Long. 30 1/2, diam. 14 mill.”; lectotype H 30.3, D 17.5, W 4.3.

##### Type material.

NHM 1975283, lectotype; 1975284, two paralectotypes (Cuming coll.).

##### Current systematic position.

Amphibulimidae, *Plekocheilus (Aeropictus) quadricolor* (Pfeiffer, 1848).

#### Bulimus rhodocheilus

Reeve, 1848

http://species-id.net/wiki/Bulimus_rhodocheilus

Figs 21E–H, 21ii

Bulimus rhodocheilus Reeve 1848 [1848–1850]: pl. 28 fig. 173.
Plekocheilus (Aeropictus) rhodocheilus  (Reeve); Breure 1978: 21, pl. 9 fig. 15 (lectotype designation); Breure 1979: 32.
Dryptus rhodocheilus  (Reeve); Simone 2006: 147, fig. 493.

##### Type locality.

“Brazil”.

##### Label.

“Brazil”. M.C. label type IV.

##### Dimensions.

Not given. Lectotype H 55.0, D 28.5, W 4.1.

##### Type material.

NHM 1975129, lectotype (Cuming coll.).

##### Remarks.

The material is accompanied by several later labels with the indication “type” or “holotype”. The specimen is damaged at the peristome. The shell is sculptured with spiral series of granules, a characteristic which accords better with *Plekocheilus (Eurytus)*. Close examination of the yellowish colour marks reveal that these are unlike the ‘air pockets' commonly found in *Plekocheilus (Aeropictus)*.

##### Current systematic position.

Amphibulimidae, *Plekocheilus (Eurytus) rhodocheilus* (Reeve, 1848) (**comb. n.**).

#### Bulimus roseolabrum

E.A. Smith, 1877

http://species-id.net/wiki/Bulimus_rhodocheilus

Figs 22D–F, 22ii

Bulimus roseolabrum  E.A. Smith, 1877: 362, pl. 39 fig. 8.
Plekocheilus (Eurytus) roseolabrum  (Smith); Breure 1978: 16 (lectotype designation); Breure 1979: 31; Breure and Borrero 2008: 6; Borrero and Breure 2011: 44, figs 13G–I.

##### Type locality.

“Malacatos, South Ecuador”.

##### Label.

“Malacatos, S. Ecuador”, in Smith' handwriting.

##### Dimensions.

“Long. 42 mill., diam 18”; lectotype H 42.0, D 22.5, W 4.5.

##### Type material.

NHM 1975135, lectotype; 1877.3.28.2, paralectotype.

##### Current systematic position.

Amphibulimidae, *Plekocheilus (Eurytus) roseolabrum* (E.A. Smith, 1877).

#### Bulimus scytodes

Pfeiffer, 1853

http://species-id.net/wiki/Bulimus_scytodes

Figs 12A–C, 12i

Bulimus scytodes Pfeiffer 1853: 256.

##### Type locality.

“in Andibus Novae Granadae”.

##### Label.

“Andes N. Granada”, taxon label in Pfeiffer's handwriting. M.C. label type I.

##### Dimensions.

“Long. 35, diam. 17 1/2 mill.”; figured specimen H 35.2, D 21.4, W 4.5.

##### Type material.

NHM 19991537, three syntypes (Cuming coll.).

##### Remarks.

This is the first time this type material is figured.

##### Current systematic position.

Amphibulimidae, *Plekocheilus (Aeropictus) veranyi* (Pfeiffer, 1848).

#### Bulimus speciosus

Pfeiffer, 1854

http://species-id.net/wiki/Bulimus_speciosus

Figs 26A–D, 26ii

Bulimus speciosus Pfeiffer 1854 [1854–1860]: 14, pl. 5 figs 1–2.
Plekocheilus (Plekocheilus) speciosus  (Pfeiffer); Breure 1978: 8 (lectotype designation); Breure 1979: 29.
Plekocheilus (Eurytus) plectostylus  (Pfeiffer); Borrero and Breure 2011: 28.

##### Type locality.

[Colombia] “Sierra Nevada de S. Marta (*Schlim*)”.

##### Label.

“Sierra Nevada de S. Marta / Schlim” [almost faded], taxon label in Pfeiffer's handwriting. M.C. label type IV.

##### Dimensions.

“Long. 58, diam. 30 mill.”; lectotype H 58.0, D 35.0, W 4.5.

##### Type material.

NHM 1975300, lectotype, Schlim leg. (Cuming coll.).

##### Remarks.

This taxon was placed in the synonymy of *Plekocheilus (Eurytus) plectostylus* (Pfeiffer, 1848) by Borrero and Breure (2011). During the prolonged time this paper was in press, the material of both taxa could be studied in the NHM. Both the size of the shell and the sculpture is markedly different (cf. Figs 20D and 25D). The previous subgeneric classification of *Plekocheilus speciosus* is thus retained.

##### Current systematic position.

Amphibulimidae, *Plekocheilus (Plekocheilus) speciosus* (Pfeiffer, 1854).

#### Bulimus superstriatus

Sowerby III, 1890

http://species-id.net/wiki/Bulimus_superstriatus

Figs 23A–C, 23i

Bulimus superstriatus Sowerby III 1890: 578, pl. 56 fig. 9.
Plekocheilus (Eurytus) superstriatus  (Sowerby); Breure 1978: 16 (lectotype designation); Breure 1979: 31.

##### Type locality.

[Peru] “Yquitos, Peruviae”.

##### Label.

“Yquitos, Peru”.

##### Dimensions.

“Long. 54, diam. 29 mill.”; lectotype H 64.5, D 31.0, W 4.8.

##### Type material.

NHM 1889.11.19.1, lectotype.

##### Remarks.

As Breure (1978) already remarked, the original dimensions of Sowerby were clearly in error.

##### Current systematic position.

Amphibulimidae, *Plekocheilus (Eurytus) superstriatus* (Sowerby III, 1890).

#### Bulimus taquinensis

Pfeiffer, 1855

http://species-id.net/wiki/Bulimus_superstriatus

Figs 26E–G, 26i

Bulimus taquinensis Pfeiffer 1855a: 290.
Plekocheilus (Eurytus) taquinensis  (Pfeiffer); Crowley and Pain 1958: 234, pl. 7 fig. 1 (lectotype designation); Breure 1979: 31.
Plekocheilus (Plekocheilus) taquinensis  (Pfeiffer); Borrero and Breure 2011: 24, figs 8G–I, 9D.

##### Type locality.

“Taquina, Sierra Nevada de S. Marta; 9000' elevation (*Schlim*)”.

##### Label.

“Sierra Nevada De S. Martha / [...] Schlim / 9000 ft high”, taxon label in Pfeiffer's handwriting. M.C. label type IV.

##### Dimensions.

“Long. 40, diam. 18 mill.”; lectotype H 40.1, D 20.5, W 4.3.

##### Type material.

NHM 1957.6.3.1, lectotype; 1957.6.3.2–3, two paralectotypes, Schlim leg.

##### Current systematic position.

Amphibulimidae, *Plekocheilus (Plekocheilus) taquinensis* (Pfeiffer, 1855).

#### Bulimus taylorianus

Reeve, 1849

http://species-id.net/wiki/Bulimus_taylorianus

Figs 24A–C, 24i

Bulimus taylorianus Reeve 1849 [1848–1850]: pl. 81 fig. 602.
Plekocheilus (Eurytus) taylorianus  (Reeve); Breure 1978: 16 (lectotype designation); Breure 1979: 31; Borrero and Breure 2011: 42, figs 15C–D.

##### Type locality.

[Ecuador] “Environs of Quito”.

##### Label.

“Quito Ecuador”.

##### Dimensions.

Not given. Lectotype H 58.5, D 31.0, W 4.7.

##### Type material.

NHM 1874.12.11.271, lectotype, ex Mus. T. Lombe Taylor.

##### Remarks.

The voucher number NHM 1975142 (Breure 1978) is here corrected to the number given above. The specimen is not accompanied by a printed label as usually found with Reeve's type material in Cuming's collection, but has a handwritten label and has reached the NHM collection via the donation by Mrs Lombe Taylor in 1875.

##### Current systematic position.

Amphibulimidae, *Plekocheilus (Eurytus) taylorianus* (Reeve, 1849).

#### Bulimus veranyi

Pfeiffer, 1848

http://species-id.net/wiki/Bulimus_veranyi

Figs 12D–F, 12ii

Bulimus veranyi Pfeiffer 1848: 230; Reeve 1848 [1848–1850]: pl. 42 fig. 262; Philippi 1849 [1847–1851]: 37, pl. 8 fig. 9.
Plekocheilus (Aeropictus) veranyi  (Pfeiffer); Breure 1978: 21, pl. 9 fig. 6 (lectotype designation); Breure 1979: 32; Borrero and Breure 2011: 12.

##### Type locality.

[Venezuela] “Chachopo, Province of Merida, New Granada (*Funck*)”.

##### Label.

“From Chachopo province / of Merida New Granada / M^r^ Funck”, taxon label in Pfeiffer's handwriting. M.C. label type IV.

##### Dimensions.

“Long. 33, diam. 15 mill.”; lectotype H 33.0, D 19.5, W 4.3.

##### Type material.

NHM 1975297, lectotype; 1975298, two paralectotypes, Funck leg. (Cuming coll.).

##### Remarks.

The ‘airpockets' typical for this subgenus are more conspicuous on the paralectotype than on the lectotype.

##### Current systematic position.

Amphibulimidae, *Plekocheilus (Aeropictus) veranyi* (Pfeiffer, 1848).

#### Plekocheilus (Aeropictus) zilchi

Breure, 1977

http://species-id.net/wiki/Plekocheilus_(Aeropictus)_zilchi

Fig. 13E

Plekocheilus (Aeropictus) zilchi Breure 1977: 260, figs 2, 21–22; Breure 1979: 32; Neubert and Janssen 2004: 235, pl. 1 fig. 5.
Plekocheilus (Aeropictus) succineoides zilchi  Breure; Borrero and Breure 2011: 17, fig. 9B.

##### Type locality.

“Colombia, Dept. Boyacá, SW Labranza grande (5°33'N, 72°35'W; 1140 m), Quebrada Comijoque”.

##### Label.

“Colombia”.

##### Dimensions.

“Shell height 40.5, diam. 25.0 (mm)”; paratype H 39.0, D 24.0, W 3.7.

##### Type material.

NHM 1975496, paratype, ex MacAndrew coll., ex Rolle.

##### Current systematic position.

Amphibulimidae, *Plekocheilus (Aeropictus) succineoides zilchi* Breure, 1977.

**Excluded from the Orthalicoidea.**

#### Bulimus elaeodes

Pfeiffer, 1853

http://species-id.net/wiki/Bulimus_elaeodes

Figs 29A–C, 29i

Bulimus elaeodes Pfeiffer 1853: 256.
Plekocheilus (Eurytus) elaeodes  (Pfeiffer); Borrero and Breure 2011: 53.

##### Type locality.

“in Andibus Novae Granadae”.

##### Label.

“Andes, N. Granada”, taxon label in Pfeiffer's handwriting. M.C. label type I.

##### Dimensions.

“Long. 36, diam. 18 mill.”; figured specimen H 33.2, D 20.6, W 4.3.

##### Type material.

NHM 19991536, three possible syntypes (Cuming coll.).

##### Remarks.

These specimens are not accompanied by a label in Pfeiffer's handwriting and their measurements do not correspond with those published by Pfeiffer. They are treated here as possible syntypes but prove not to belong to the genus *Plekocheilus* to which this taxon was hitherto referred.

##### Current systematic position.

Strophocheilidae, *Chileborus* species?.

**Figure 7.**
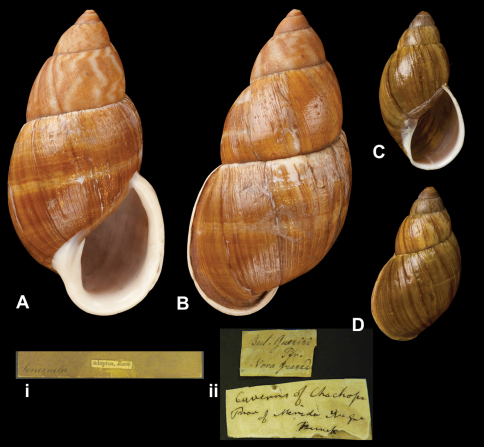
*Dryptus* species. **A–B, i** *Dryptus marmoratus* (Dunker, 1844), lectotype of *Bulimus adoptus* Reeve, 1849 NHM 20100517 (H = 83.3) **C–D, ii** *Dryptus gueirini* (Pfeiffer, 1846), lectotype NHM 1975272 (H = 41.0).

**Figure 8.**
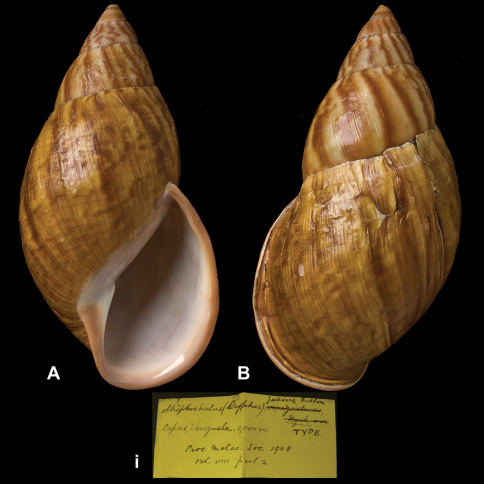
**A–B, i** *Dryptus jubeus* (Fulton, 1908), lectotype NHM 1905.5.3.1 (H = 117.5).

**Figure 9.**
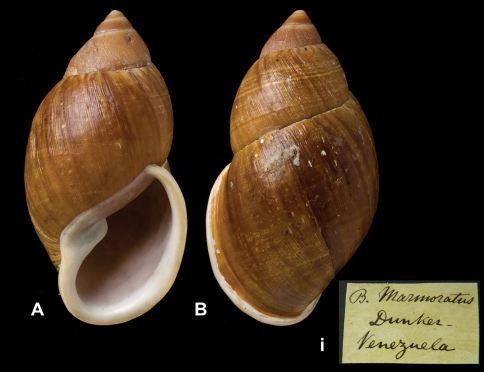
**A–B, i** *Dryptus marmoratus* (Dunker, 1844), lectotype NHM 1975474 (H = 86.1).

**Figure 10.**
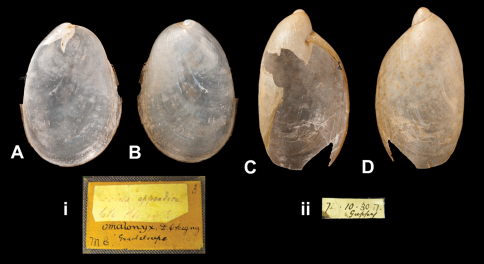
**A–B, i** *Pellicula appendiculata* (Pfeiffer, 1847), syntype NHM 20110303 (H = 14.2) **C–D, ii** *Amphibulima pardalina* Guppy, 1868, lectotype NHM 1874.10.30.7 (H = 18.9).

**Figure 11.**
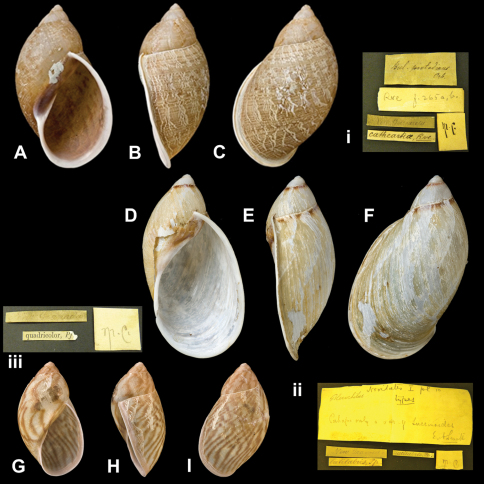
*Plekocheilus (Aeropictus)* species. **A–C, i** *Plekocheilus (Aeropictus) cathcartiae* (Reeve, 1848), lectotype NHM 1975288 (H = 45.4) **D–F, ii** *Plekocheilus (Aeropictus) latilabris* (Pfeiffer, 1855), lectotype NHM 1975127 (H = 49.0) **G–I, iii** *Plekocheilus (Aeropictus) quadricolor* (Pfeiffer, 1848), lectotype NHM 1975283 (H = 30.3).

**Figure 12.**
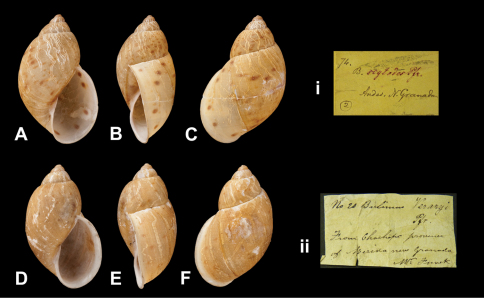
*Plekocheilus (Aeropictus)* species. **A–C, i** *Plekocheilus (Aeropictus) veranyi* (Pfeiffer, 1848), syntype of *Bulimus scytodes* Pfeiffer, 1853 NHM 19991537 (H = 35.2) **D–F, ii** *Plekocheilus (Aeropictus) veranyi* (Pfeiffer, 1848), lectotype NHM 1975297 (H = 33.0).

**Figure 13.**
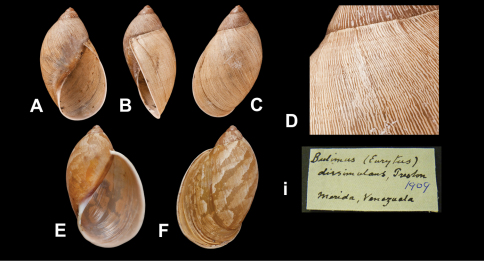
*Plekocheilus (Aeropictus)* species.  **A–D, i** *Plekocheilus (Aeropictus) dissimulans* Preston, 1909, lectotype NHM 1914.4.3.37 (H = 30.0) **E–F** *Plekocheilus (Aeropictus)* zilchi Breure, 1977, paratype NHM 1975496 (H = 39.0).

**Figure 14.**
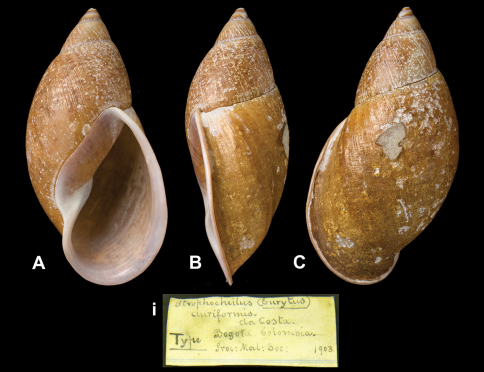
**A–C, i** *Plekocheilus (Eurytus) auriformis* (da Costa, 1904), holotype NHM 1907.11.21.112 (H = 74.0)

**Figure 15.**
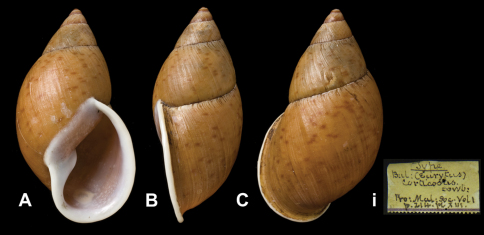
**A–C, i** *Plekocheilus (Eurytus) episcopalis corticosus* (Sowerby III, 1895), lectotype NHM 1907.11.21.110 (H = 58.7).

**Figure 16.**
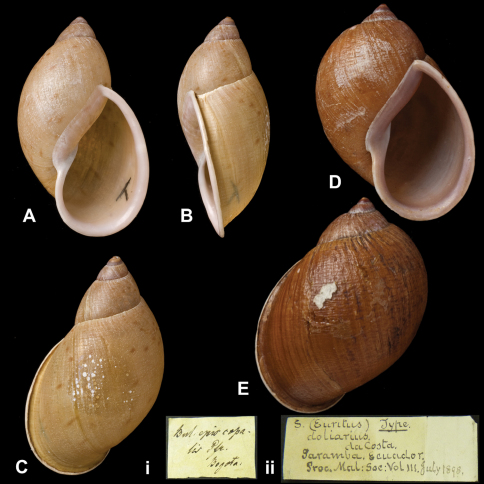
*Plekocheilus (Eurytus)* species. **A–C, i** *Plekocheilus (Eurytus) episcopalis episcopalis* (Pfeiffer, 1855), lectotype NHM 1953.11.30.1 (H = 58.0) **D–E, ii** *Plekocheilus (Eurytus) doliarius* (da Costa, 1898), lectotype NHM 1907.11.21.117 (H = 58.0).

**Figure 17.**
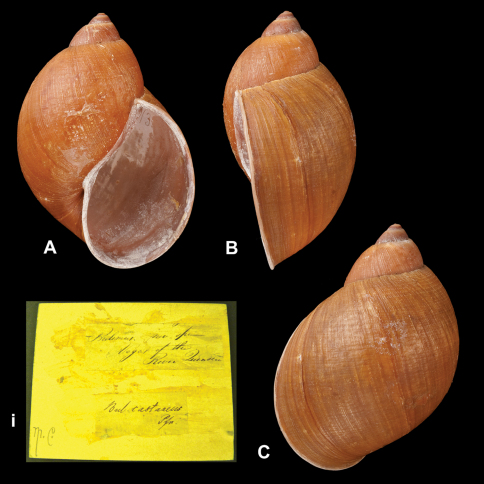
**A–C, i** *Plekocheilus (Eurytus) castaneus* (Pfeiffer, 1845), lectotype NHM 1975279 (H = 69.5).

**Figure 18.**
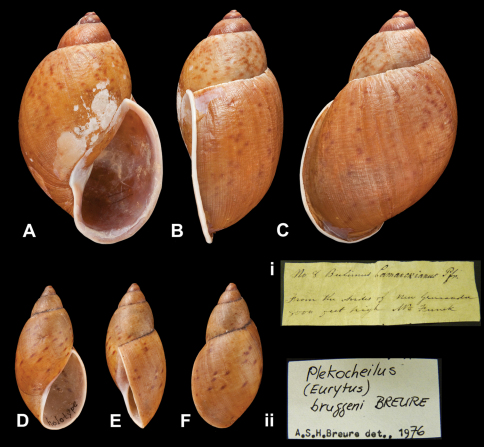
*Plekocheilus (Eurytus)* species. **A–C, i** *Plekocheilus (Eurytus) lamarckianus* (Pfeiffer, 1848), lectotype NHM 1975259 (H = 62.4) **D–F, ii** *Plekocheilus (Eurytus) bruggeni* Breure, 1978, holotype NHM 1911.11.2.88 (H = 39.0).

**Figure 19.**
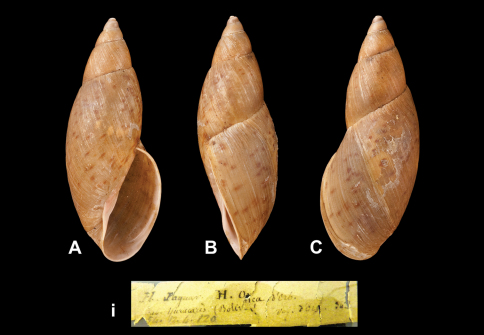
**A–C, i** *Plekocheilus (Eurytus) pentadinus* (d'Orbigny, 1835), lectotype of *Helix onca* d'Orbigny, 1835 (H = 66.5).

**Figure 20.**
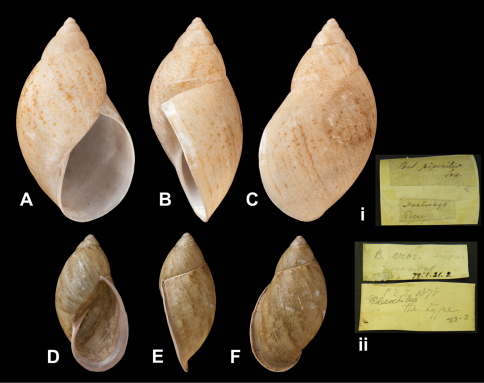
*Plekocheilus (Eurytus)* species. **A–C, i** *Plekocheilus (Eurytus) piperitus* (Sowerby I, 1837), syntype NHM 1975329 (H = 55.8) **D–F, ii** *Plekocheilus (Eurytus) eros* (Angas, 1878), lectotype NHM 1879.1.21.2 (H = 35.5).

**Figure 21.**
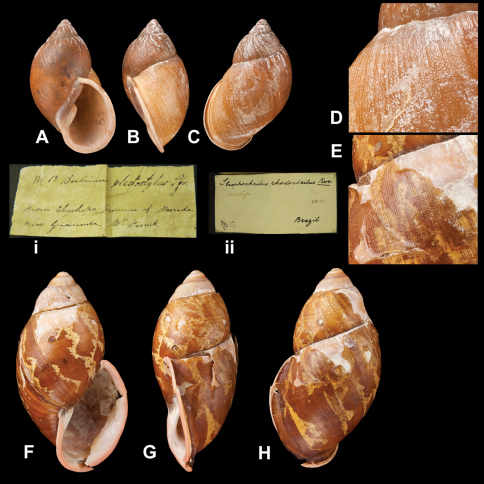
*Plekocheilus (Eurytus)* species. **A–D, i** *Plekocheilus (Eurytus) plectostylus* (Pfeiffer, 1848), lectotype NHM 1975287 (H = 36.0); **D** sculpture of dorsal side of last whorl **E–H, ii** *Plekocheilus (Eurytus) rhodocheilus* (Reeve, 1848), lectotype NHM 1975129 (H = 55.0); **E** sculpture of dorsal side of last whorl.

**Figure 22.**
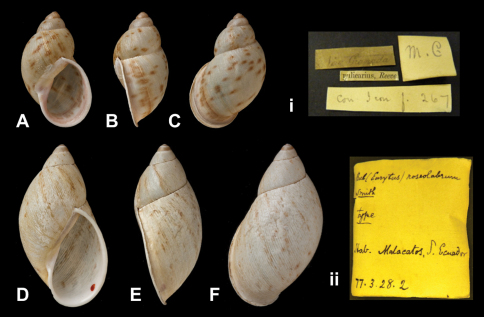
*Plekocheilus (Eurytus)* species. **A–C, i** *Plekocheilus (Eurytus) pulicarius* (Pfeiffer, 1848), lectotype NHM 1975281 (H = 31.5) **D–F, ii** *Plekocheilus (Eurytus) roseolabrum* (E.A. Smith, 1877), lectotype NHM 1975135 (H = 42.0).

**Figure 23.**
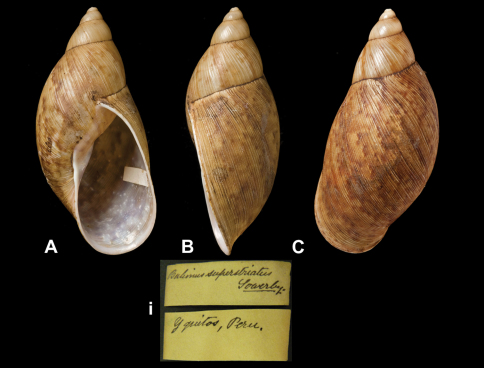
**A–C, i** *Plekocheilus (Eurytus) superstriatus* (Sowerby III, 1890), lectotype NHM 1889.11.19.1 (H = 64.5).

**Figure 24.**
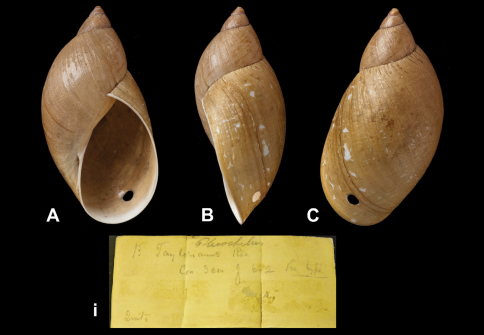
**A–C, i** *Plekocheilus (Eurytus) taylorianus* (Reeve, 1849), lectotype NHM 1874.12.11.271 (H = 58.5).

**Figure 25.**
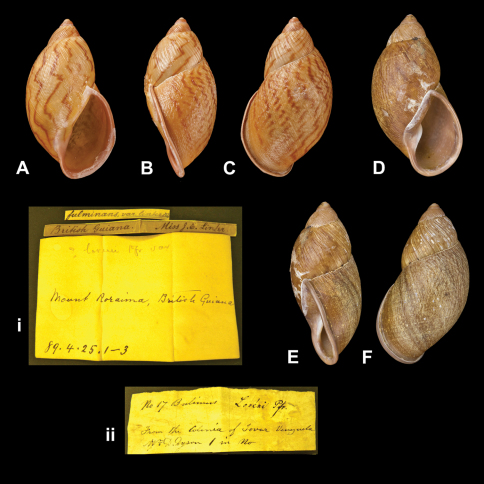
*Plekocheilus (Plekocheilus)* species. **A–C, ii** *Plekocheilus (Plekocheilus) loveni* (Pfeiffer, 1848), lectotype NHM 1975285 (H = 43.5) **D–F, i** *Plekocheilus (Plekocheilus) linterae* (Sowerby III, 1890), lectotype NHM 1889.4.25.1 (H = 43.8).

**Figure 26.**
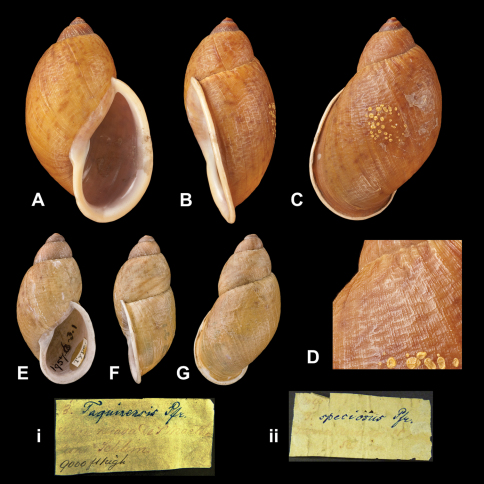
*Plekocheilus (Plekocheilus)* species. **A–D, ii** *Plekocheilus (Plekocheilus) speciosus* (Pfeiffer, 1855), lectotype NHM 1975300 (H = 58.0); **D** sculpture of dorsal side of last whorl **E–G, i** *Plekocheilus (Plekocheilus) taquinensis* (Pfeiffer, 1855), lectotype NHM 1957.6.3.1 (H = 40.1).

**Figure 27.**
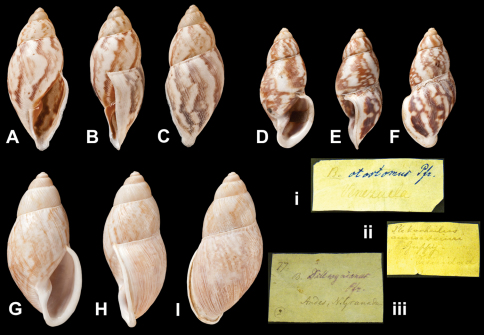
*Plekocheilus (Eudolichotis)* species **A–C, ii** *Plekocheilus (Eurytus) aurissciuri* Guppy, 1866, syntype NHM 1866.1.3.6 (H = 36.6) **D–F, i** *Plekocheilus (Eurytus) otostomus* (Pfeiffer, 1855), lectotype NHM 1975307 (H = 31.5) **G–I, iii** *Plekocheilus (Eurytus) dillwynianus* (Pfeiffer, 1853), lectotype NHM 1975144 (H = 39.5).

**Figure 28.**
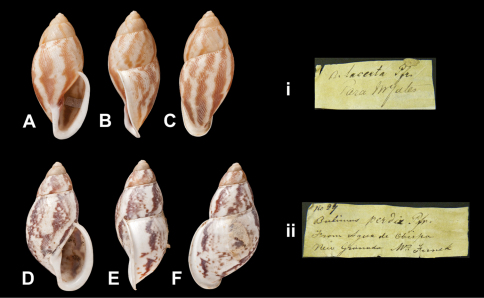
*Plekocheilus (Eudolichotis)* species A–C, i P*. (E.) lacerta* (Pfeiffer, 1855), lectotype NHM 1975303 (H = 33.5) D–F, ii *Plekocheilus (Eurytus) perdix* (Pfeiffer, 1848), lectotype NHM 1975305 (H =  33.5).

**Figure 29.**
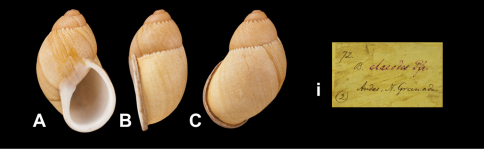
*Chileborus* species. **A–C, i** Syntype of *Bulimus elaeodes* Pfeiffer, 1853 NHM 19991536 (H = 33.2).

## Supplementary Material

XML Treatment for Bulimus adoptus

XML Treatment for Succinea appendiculata

XML Treatment for Strophocheilus (Eurytus) auriformis

XML Treatment for Plekocheilus aurissciuri

XML Treatment for Plekocheilus (Eurytus) bruggeni

XML Treatment for Bulimus castaneus

XML Treatment for Bulimus cathcartiae

XML Treatment for Bulimus (Eurytus) corticosus

XML Treatment for Bulimus dillwynianus

XML Treatment for Bulimus (Eurytus) dissimulans

XML Treatment for Strophocheilus (Eurytus) doliarius

XML Treatment for Bulimus episcopalis

XML Treatment for Bulimus (Eurytus) eros

XML Treatment for Bulimus guerini

XML Treatment for Strophocheilus (Dryptus) jubeus

XML Treatment for Bulimus lacerta

XML Treatment for Bulimus lamarckianus

XML Treatment for Bulimus latilabris

XML Treatment for Bulimus fulminans linterae

XML Treatment for Bulimus loveni

XML Treatment for Bulimus marmoratus

XML Treatment for Helix onca

XML Treatment for Bulimus otostomus

XML Treatment for Amphibulima pardalina

XML Treatment for Bulimus perdix

XML Treatment for Bulinus piperitus

XML Treatment for Bulimus plectostylus

XML Treatment for Bulimus pulicarius

XML Treatment for Bulimus quadricolor

XML Treatment for Bulimus rhodocheilus

XML Treatment for Bulimus roseolabrum

XML Treatment for Bulimus scytodes

XML Treatment for Bulimus speciosus

XML Treatment for Bulimus superstriatus

XML Treatment for Bulimus taquinensis

XML Treatment for Bulimus taylorianus

XML Treatment for Bulimus veranyi

XML Treatment for Plekocheilus (Aeropictus) zilchi

XML Treatment for Bulimus elaeodes
